# Overexpression of GmPAP4 Enhances Symbiotic Nitrogen Fixation and Seed Yield in Soybean under Phosphorus-Deficient Condition

**DOI:** 10.3390/ijms25073649

**Published:** 2024-03-25

**Authors:** Xi Sun, Huantao Zhang, Zhanwu Yang, Xinzhu Xing, Zhao Fu, Xihuan Li, Youbin Kong, Wenlong Li, Hui Du, Caiying Zhang

**Affiliations:** 1 State Key Laboratory of North China Crop Improvement and Regulation, Hebei Agricultural University, Baoding 071000, China; 20212100284@pgs.hebau.edu.cn (X.S.); 20222100267@pgs.hebau.edu.cn (H.Z.); zhanwuyang89@126.com (Z.Y.); xxinzhu@pgs.hebau.edu.cn (X.X.); 20232100272@pgs.hebau.edu.cn (Z.F.); lixihuan@hebau.edu.cn (X.L.); kong_1985@163.com (Y.K.); wenlongli82@126.com (W.L.); 2 North China Key Laboratory for Crop Germplasm Resources of Education Ministry, College of Agronomy, Hebei Agricultural University, Baoding 071000, China

**Keywords:** symbiosis, nitrogen-fixing rhizobia, biological nitrogen fixation, purple acid phosphatase, phosphorus stress

## Abstract

Legume crops establish symbiosis with nitrogen-fixing rhizobia for biological nitrogen fixation (BNF), a process that provides a prominent natural nitrogen source in agroecosystems; and efficient nodulation and nitrogen fixation processes require a large amount of phosphorus (P). Here, a role of GmPAP4, a nodule-localized purple acid phosphatase, in BNF and seed yield was functionally characterized in whole transgenic soybean (*Glycine max*) plants under a P-limited condition. *GmPAP4* was specifically expressed in the infection zones of soybean nodules and its expression was greatly induced in low P stress. Altered expression of *GmPAP4* significantly affected soybean nodulation, BNF, and yield under the P-deficient condition. Nodule number, nodule fresh weight, nodule nitrogenase, APase activities, and nodule total P content were significantly increased in *GmPAP4* overexpression (OE) lines. Structural characteristics revealed by toluidine blue staining showed that overexpression of *GmPAP4* resulted in a larger infection area than wild-type (WT) control. Moreover, the plant biomass and N and P content of shoot and root in *GmPAP4* OE lines were also greatly improved, resulting in increased soybean yield in the P-deficient condition. Taken together, our results demonstrated that GmPAP4, a purple acid phosphatase, increased P utilization efficiency in nodules under a P-deficient condition and, subsequently, enhanced symbiotic BNF and seed yield of soybean.

## 1. Introduction

Legume plants, such as the common bean (*Phaseolus vulgaris*), soybean (*Glycine max*), pea (*Pisum sativum*), and chickpea (*Cicer arietinum* L.), are very important legume crops that provide humans and animals with food and feed materials. Legume plants can establish symbiotic associations with nitrogen (N)-fixing rhizobia in soil [1,2]. Symbiotic biological nitrogen fixation (BNF) is processed in nodules and the resulting fixed nitrogen products are in the form of ureides, allantoin, and allantoic acids transported from nodules to shoot for plant growth and development [3,4,5]. In order to meet the food demand for all the population of the world, researchers aim to improve crop production; however, agriculture is highly dependent on the application of excessive industrial nitrogen fertilizer in the field, which not only costs a heavy price, and reduces the utilization efficiency of nitrogen fertilizer, but also results in production reduction, waste of resources, lower environment impacts, and so on [6,7]. Thus, it is of significance to find a natural way of replacement of nitrogen fertilizer and increase nitrogen utilization efficiency. For this purpose, legume plants are of great interest due to their capacity for nitrogen fixation.

Although nitrogen fixation in nodules benefits soybean plants with N nutrient, the nodulation and nitrogen fixation process consumes much energy. Several studies have reported that P was particularly essential for nodulation and energy consumption for N_2_ fixation in legume plants. P deficiency severely inhibits nodule development, nitrogen fixation, and total N content in legume plants [8,9]. In *Medicago truncatula*, under P-depleted condition, nodule dry matter (DM) and the resulting total concentration of amino-N compounds in nodules was significantly reduced compared to the normal P growth condition [10]. In soybean, the nodule number, nodule fresh weight and size, and the nitrogenase activity decreased significantly in low P-stress condition [11,12]. Therefore, legume plants have developed several adaptive strategies to maintain P homeostasis for symbiotic nitrogen fixation in nodules under P-starvation condition, including induction and secretion of acid phosphatase (APase; EC 3.1.3.2) activity of roots and nodules [13,14].

Purple acid phosphatases (PAPs; EC 3.1.3.2), a specific group of acid phosphatases, have been found in several plant species, such as *Arabidopsis thaliana*, common bean (*Phaseolus vulgaris*), rice *(Oryza sativa*), *Medicago truncatula*, and soybean [15,16,17,18]. PAPs have diverse functions in plants and most of them were induced by P starvation. PvPAP3, in common bean (*Phaseolus vulgaris*), was induced in P starvation and enhanced utilization efficiency of extracellular ATP supplied as the sole P source [18]. AtPAP12 and AtPAP26 in *Arabidopsis thaliana* were identified as the predominant PAP isozymes secreted by roots under P-deficient condition and played a vital role in improving extracellular P-use efficiency [15]. *GmPAP7a/7b* were also up-regulated by P starvation and data indicated that *GmPAP7a/7b* contributed to extracellular ATP utilization in soybean [19]. *GmPAP17* had a strong response to low phosphorus stress in root functions in the adaptation of soybean to low P stress, possibly through its involvement in P recycling in plants [20]. All these published research papers suggested that PAP members could be involved in P acquisition and recycling in plants.

However, not so many research papers focused on the role of PAPs in nodule development and nitrogen fixation in legume plants under P-deficient condition. Nodulin PvNOD33, a putative phosphatase from the common bean, was highly expressed in the inner cortex of infection cells of mature nodules and was inferred to function in carbon metabolism [21]. A novel PAP gene, *GmPAP21*, in soybean, was greatly induced by P limitation in soybean nodules, and altered expression of *GmPAP21* significantly affected acid phosphatase and nodule growth and development [22]. Another PAP gene, *GmPAP12*, reported by our team previously, was highly expressed in nodules and promoted nodule development and nitrogen fixation by enhancing P utilization under P-limitation condition [14]. Previously, our research team found that *GmPAP4*, a new purple acid phosphatase, showed highly induced expression in soybean roots under P-deficient conditions, and overexpression of *GmPAP4* in Arabidopsis resulted in significant rises in P acquisition and utilization compared with the wild-type control [17]. Here, we reported that *GmPAP4* was also highly expressed in nodules during development in soybean and its expression was induced by low P stress. Systematical experiments were further conducted to explore the function of *GmPAP4* in nodule development and nitrogen fixation under P-deficient condition. We found that overexpression of *GmPAP4* significantly increased nodule development and nitrogen fixation, and, finally, improved plant growth and seed yield under P-deficient condition.

## 2. Results

### 2.1. GmPAP4 Was Preferentially Expressed in Infection Zones of Nodules in Soybean

**Figure 1:**
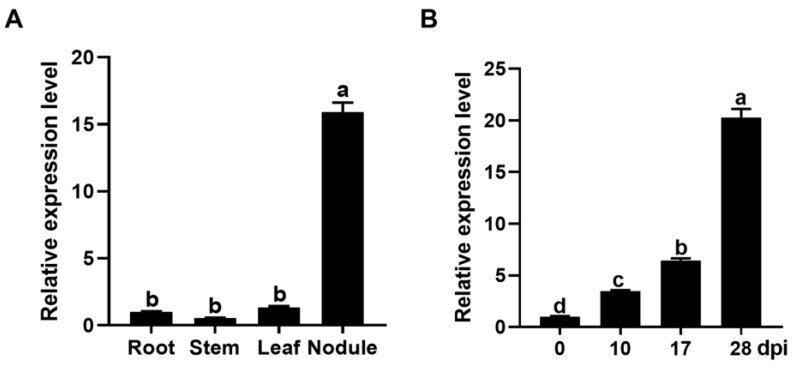
Spatio-temporal relative expression of *GmPAP4* in soybean. (**A**) Expression profiles of *GmPAP4* in various organs of soybean. (**B**) Time course expression analysis of *GmPAP4* in nodules (0, 10, 17, and 28 dpi). The lowercase letters a–d indicate significant differences, *p* < 0.05.

To investigate the expression pattern, the transcript abundance of *GmPAP4* was examined by quantitative real-time PCR (qRT-PCR) in various organs of soybean inoculated with rhizobia in low-nitrogen nutrient condition. We found that *GmPAP4* was expressed higher in nodules than in any other organs and expression of *GmPAP4* was increased during nodule development, suggesting a role of *GmPAP4* in soybean nodulation (Figure 1).

**Figure 2:**
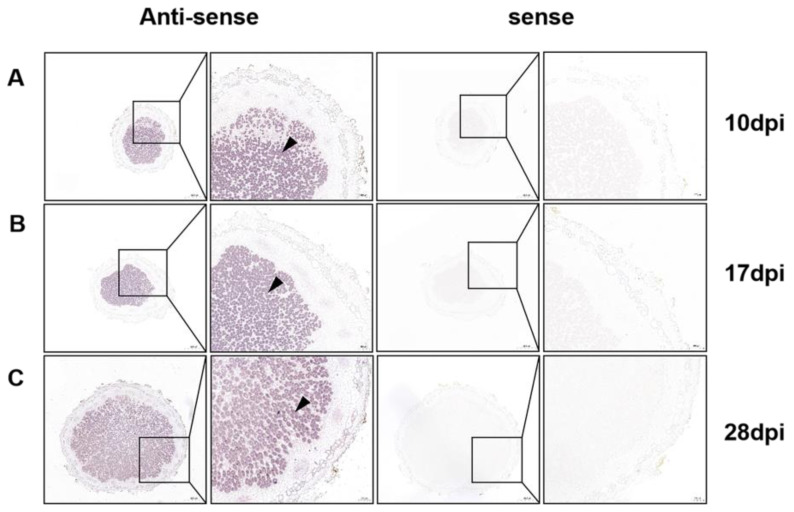
RNA in situ *hybridization* analysis of *GmPAP4* in different developmental stages of nodules. Soybean nodules were harvested at 10, 17, and 28 dpi (**A**–**C**) and digoxigenin-labelled probes specific for *GmPAP4* were used for the detection of its transcript. The black arrow head indicates the infected cells in the nodules. Scale bar = 500 µm (left panel of antisense or sense) and 100 µm (right panel of antisense or sense).

Next, to precisely determine the tissue-specific localization of *GmPAP4*, we carried out RNA in situ *hybridization* analysis in different developmental stages of nodules. We found that the transcript of *GmPAP4* was specifically localized in the nitrogen-fixing zones of nodules in all the developmental stages. There was no hybridization signal detected in nodule samples with a sense probe, suggesting the specificity of the antisense probe (Figure 2). Together, these results demonstrate that *GmPAP4* genes potentially function in nodule development and nitrogen fixation.

### 2.2. GmPAP4 Was Highly Induced by Low Phosphorus Stress in Soybean Nodules

**Figure 3:**
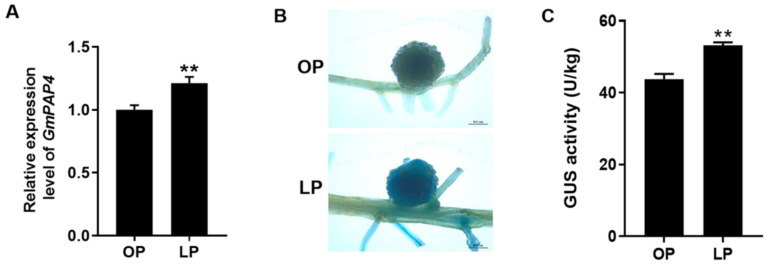
Response of *GmPAP4* to LP stress. (**A**) The relative expression value of *GmPAP4* in nodules under different P conditions. The expression value of *GmPAP4* was normalized based on the expression of *GmActin11* (*Glyma.18g290800*) used as a reference gene. (**B**) Histochemical staining analysis of the promoter of *GmPAP4* in transgenic composite soybean roots and nodules at 28 dpi, Scale bar = 0.1 cm. (**C**) GUS activity of nodules expressing p*GmPAP4*:GUS. Three independent experiments were performed and images from one representative experiment were shown here (n > 10). Each error bar represents the mean of three biological replicates with ±SE. Asterisks indicate significant differences within a *p* level in *t*-tests (**: 0.001 < *p* ≤ 0.01).

To determine the response of *GmPAP4* to low phosphorus (LP) stress in nodules, we planted the soybean plants inoculated with rhizobia under optimal phosphorus (OP) or LP conditions for 28 days. Our qRT-PCR analysis showed that *GmPAP4* could be induced under LP stress compared to OP condition (Figure 3A). To further elucidate the expression of *GmPAP4* in nodules under different P conditions, we cloned the promoter of *GmPAP4* 2000 bp upstream of the translational start codon fused with the GUS reporter gene (p*GmPAP4*:GUS). And the transgenic composite plants carrying p*GmPAP4*:GUS were inoculated with rhizobia and the transgenic roots and nodules were harvested at 28 days post inoculation (dpi) for GUS staining under optimal phosphorus (OP) and LP conditions. The results showed that the promoter of *GmPAP4* was highly expressed in nodules and GUS activity was increased by 21.6% under LP stress compared with OP condition (Figure 3B,C). All these data indicated that *GmPAP4* may be involved in P signaling in nodules of soybean.

### 2.3. GmPAP4 Expression Was Positively Associated with Nodulation and Nitrogen Fixation under LP Stress

To further investigate the role of *GmPAP4* in nodulation and biological nitrogen fixation under LP stress, stable transgenic soybean plants overexpressing *GmPAP4* were generated through *Agrobacterium*-mediated transformation. Finally, two stable transgenic overexpression lines (OE1 and OE2) were obtained and confirmed by specific PCR and glyphosate resistance tests (Supplemental Figure S1). RT-PCR analysis showed that the transcript accumulation of *GmPAP4* was about 10 times higher in OE lines than in nodules of WT control and the expression level of *GmPAP4* in nodules of the two stable lines did not show significant differences (Supplemental Figure S2). Next, the structural characteristics of nodules formed on *GmPAP4* OE lines were examined. Images of cross-sectioned nodules stained with toluidine blue dye revealed that *GmPAP4* overexpression greatly affected nodule morphological development (Supplemental Figure S3A). In comparison with WT nodules, the two *GmPAP4* OE lines displayed larger infection zones by 17.0% and 14.4%, respectively (Supplemental Figure S3B). These results further indicated that *GmPAP4* was associated with nodule formation and, therefore, was crucial for nitrogen fixation.

**Figure 4:**
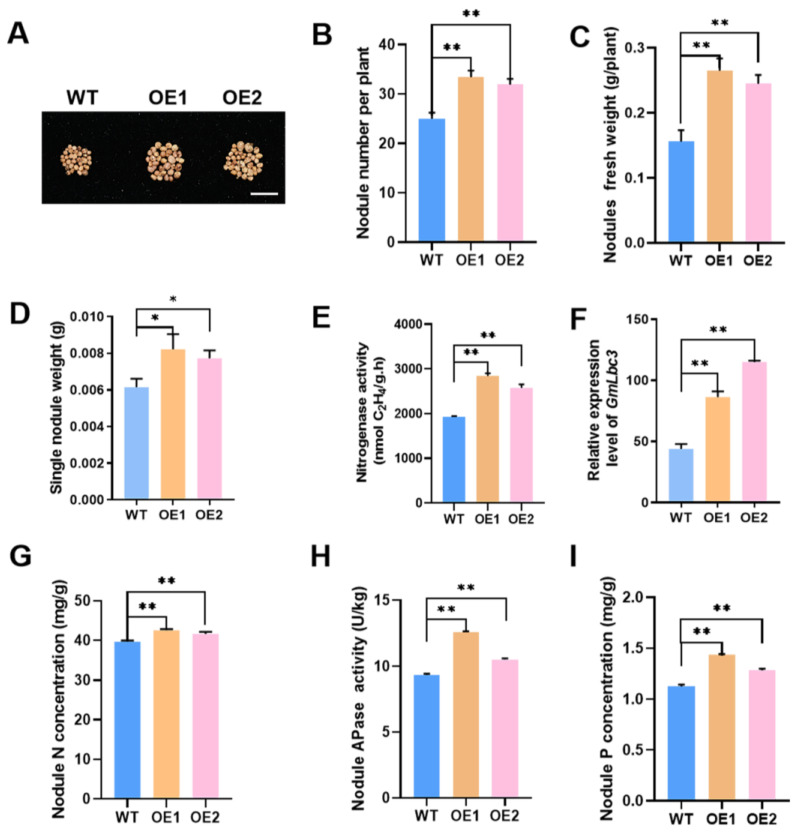
Phenotypic analysis of nodulation of stable transgenic soybean plants overexpressing (OE) of *GmPAP4*. (**A**) Nodule growth performance at 28 dpi. Scale bar in (**A**) = 1 cm. (**B**) Nodule number per plant. (**C**) Nodule fresh weight. (**D**) Single nodule weight. (**E**) Nitrogenase activity measured by the acetylene reduction assay. (**F**) Quantitative real-time PCR analysis of *GmLbc3* expression in nodules. (**G**) Nodule N concentration. (**H**) APase activities of nodules. (**I**) Nodule P concentration. Each error bar represents the mean of three biological replicates with ±SE. Asterisks indicate significant differences within a *p* level in *t*-tests (*: 0.01 < *p* ≤ 0.05, **: 0.001 < *p* ≤ 0.01).

Subsequent evaluation was conducted to determine the effect of altered expression of *GmPAP4* on nodule development and growth under LP condition at 28 dpi, at which point the nodules were more functional and active. We found that overexpression of *GmPAP4* increased nodule development and biological nitrogen fixation ability. The two stable transgenic lines (OE1 and OE2) showed an increase of 33.6% and 27.6% in the total nodule number, an increase of 69.9% and 57.1% in nodule fresh weight, resulting in increased signal nodule weight, and an increase of 47.8% and 32.7% in nitrogenase activity, respectively (Figure 4A–E). On the other hand, the functional and active nodules were always pink due to the presence of the leghemoglobin (GmLbc3) protein, an oxygen carrier, required for nitrogenase activity and biological nitrogen fixation in nodules [23]. We found that the expression of *GmLbc3* in nodules of two *GmPAP4* OE lines was greatly increased compared with that in WT nodules (Figure 4F). And, finally, the nodule N concentration in the two stable lines showed a 7.2% and 5.0% increase compared with WT control (Figure 4G). All these data suggested that overexpression of *GmPAP4* directly increased nodule development as well as the capacity of symbiotic nitrogen fixation in nodules of soybean under LP condition.

Next, to confirm whether the improved nodulation in *GmPAP4* OE lines is due to overexpression of *GmPAP4*, APase activities of nodules in *GmPAP4* OE lines were assessed under low P condition and we found that overexpression of *GmPAP4* led to 34.6% and 12.6% increases in nodule APase activities relative to WT control (Figure 4H). Consequently, the P concentration of nodules was enhanced significantly, with 27.5% and 14.1% increases in the two *GmPAP4* OE lines (Figure 4I). These data indicated that *GmPAP4* might facilitate nodule development and nitrogen fixation by enhancing P utilization efficiency in nodules of soybean under P-deficient condition.

### 2.4. Overexpression of GmPAP4-Enhanced Soybean Plant Growth under P-Deficient Conditions

**Figure 5:**
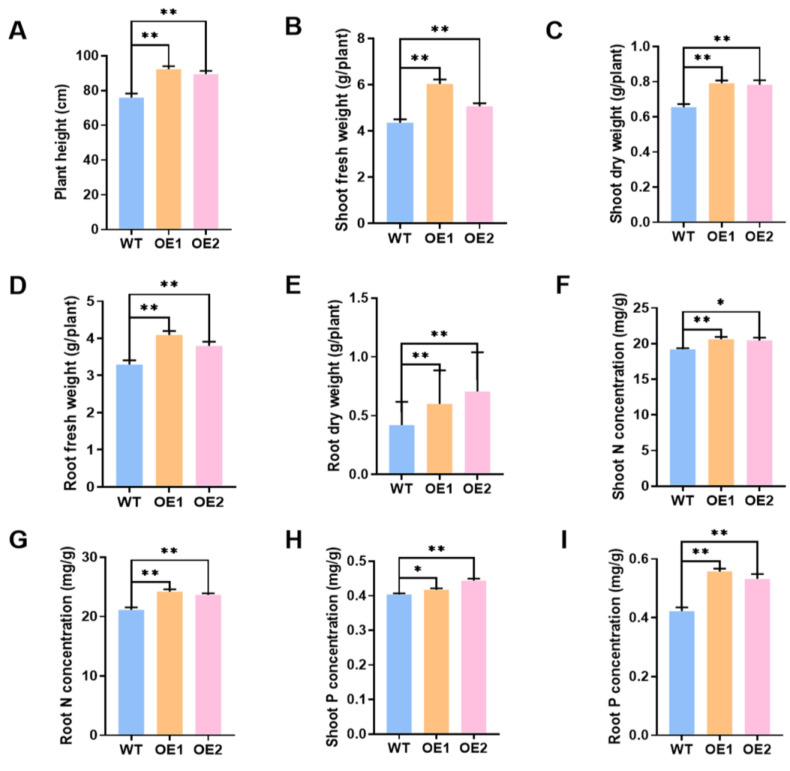
Phenotypic analysis of stable transgenic soybean plants overexpressing of *GmPAP4* at 28 dpi under low P stress. (**A**) Plant height. (**B**) Shoot fresh weight. (**C**) Shoot dry weight. (**D**) Root fresh weight. (**E**) Root dry weight. (**F**) Shoot N concentration. (**G**) Root N concentration. (**H**) Shoot P concentration. (**I**) Root P concentration. Each error bar represents the mean of three biological replicates with ±SE. Asterisks indicate significant differences within a *p* level in *t*-tests. (*: 0.01 < *p* ≤ 0.05, **: 0.001 < *p* ≤ 0.01).

In the meantime, the role of overexpression of *GmPAP4* on soybean plant growth inoculated with rhizobia was evaluated under LP condition and we found that overexpression of *GmPAP4* resulted in much better growth relative to the control plants. Overexpression of *GmPAP4* led to 21.5% and 17.7% increases in plant height, 38.7% and 16.2% increases in shoot fresh weight, and 20.7% and 13.2% increases in shoot dry weight, respectively. In the case of soybean roots, we found that root fresh weight increased by 24.3% and 15.4% and root dry weight increased by 43.4% and 24.8% in *GmPAP4* OE lines (Figure 5A–E). Moreover, the two transgenic lines showed 7.4% and 6.6% increases in N content of the shoots and 14.6% and 12.1% increases in N content of the roots, and increases of 3.3% and 9.9% and 32.1% and 26.1% in P content of the shoots and roots, respectively, relative to the control plants (Figure 5F–I).

### 2.5. Overexpression of GmPAP4 Enhanced Soybean Yield under P-Deficient Conditions

**Figure 6:**
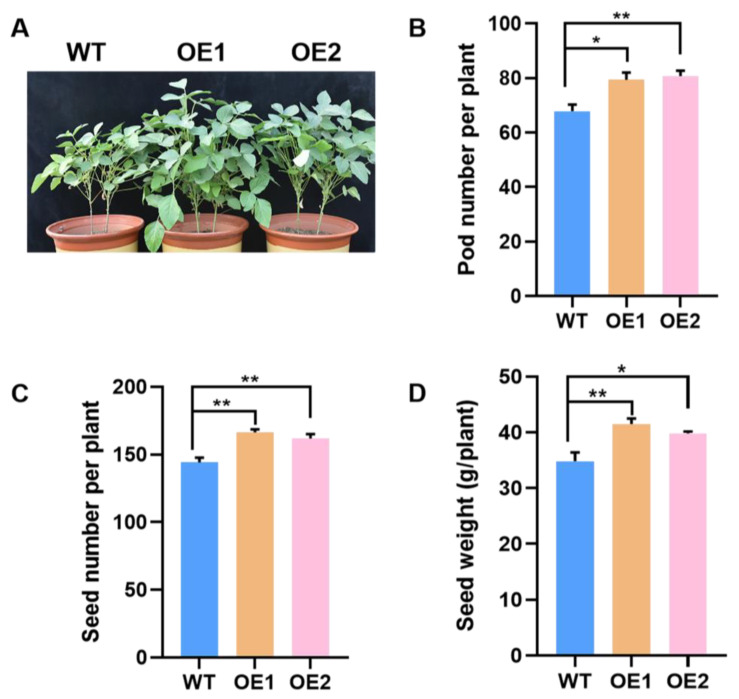
Seed yield analysis of *GmPAP4* OE lines in the pot experiment under low P condition. (**A**) Plant growth performance at pot culture. (**B**) Pod number per plant. (**C**) Seed number per plant. (**D**) Seed weight per plant. Each error bar represents the mean of three biological replicates with ±SE. Asterisks indicate significant differences within a *p* level in *t*-tests (*: 0.01 < *p* ≤ 0.05, **: 0.001 < *p* ≤ 0.01).

The growth performance of *GmPAP4* OE lines subsequently led to higher seed yield compared with the WT control. *GmPAP4* OE lines resulted in increases in pod number by 17.0% and 19.0%, increases in seed number by 15.4% and 12.3%, and increases in seed weight by 19.2% and 14.4%, respectively (Figure 6). All these results suggested that *GmPAP4* was associated with nodule formation and nitrogen fixation and, subsequently, affected soybean growth, N nutrition, and grain yield under P-deficient stress.

## 3. Discussion

Symbiotic BNF in root nodules plays a crucial role in sustainable agricultural systems and it is an effective alternative way of chemical N fertilizer to provide N nutrient for legume plants [24]. Many studies demonstrate that P is particularly critical in the rhizobium–legume symbiotic process, not only due to its regulation in nodule development but also due to the energy costs of N fixation [25,26]. Several genes, such as purple acid phosphatases, β-expansins, phosphate transporters, GmSPX members, and some transcription factors (GmPHR1, GmPTF1) in soybean, were reported to be highly upregulated in nodules under low P stress [12,27,28,29]. In the soybean whole genome, a total of 38 GmPAPs were isolated and many of the members in the leaves and roots were upregulated at 16 days of P deficiency [19]. However, the functions of most low P-starved up-regulated GmPAPs in nodules, except of GmPAP12 and GmPAP21, remain largely understood [14,22].

In the present study, we found *GmPAP4* was expressed highly and specifically in infection zones of nodules detected by qRT-PCR and RNA in situ *hybridization* (Figure 1 and Figure 2) and GUS activity driven by the promoter of *GmPAP4* in composite transgenic nodules was increased significantly in response to P deficiency, indicating the involvement of *GmPAP4* in P uptake and utilization for nodule development and nitrogen fixation (Figure 3). Previously, for tissue localization of GmPAP12 and GmPAP21, only RT-PCR and promoter-GUS staining experiments were conducted and they found *GmPAP12* and *GmPAP21* were highly expressed in the organs of nodules [14,22]. Here, RNA in situ *hybridization* was performed to specify the infection zone localization of *GmPAP4* inside nodules, making the hypothesis of the important role of *GmPAP4* in nodule development more reliable and accurate.

Previously, to study the function of *GmPAP12* in nodule development and nitrogen fixation, overexpressed composite transgenic lines were generated and evaluated. In the present study, in order to be more convincing, stable transgenic overexpression lines were conducted for the role of *GmPAP4* under low P condition (Supplemental Figure S1). The two OE lines exhibited more increased nodule development and nitrogen fixation efficiency (Figure 4 and Figure 5). In the adaptation of the symbiosis process to P starvation, legumes can enhance P utilization within the nodules to tolerate P deficiency [30]. In this study, we found that *GmPAP4*-OE lines increase APase activity and total P and N acquisition, suggesting that *GmPAP4* overexpression enhanced biological nitrogen fixation by more P acquisition (Figure 5). These findings were consistent with the results observed by the overexpression of *GmPAP12* under P deficiency. While overexpression of *GmPAP21* reduced nodule dry weight, P and N contents in nodules of stable transgenic plants showed contrary results with *GmPAP4*. All these results indicated that different GmPAP members may cooperate in nodulation by balancing the P acquisition and nitrogen fixation efficiency in soybean nodules.

In the meantime, we found that the two OE lines improved not only nodule development and nitrogen fixation but also plant development and seed yield under P-stress condition (Figure 6). This might be due to the contributions of enhanced N and P acquisition and plant development through symbiotic nitrogen fixation. Taken together, the functional study results presented here strongly suggest that *GmPAP4* was involved in plant growth and development regulation that was realized through increased nitrogen and phosphate efficiency, and it played a role in the cross-talk between nitrogen and phosphate pathways in soybean nodules.

## 4. Materials and Methods

### 4.1. Plant Materials and Growth Conditions

The soybean (*Glycine max* (L.) *Merr.*) seeds used here were originally obtained from the State Key Laboratory for North China Crop Improvement and Regulation, Hebei Agricultural University. Soybean cultivar Jidou12 and two *GmPAP4* overexpression lines in the Jidou12 background were used for phenotypic and functional analysis in all experiments in this study.

Soybean seeds were planted in the vermiculite for 5 days in a controlled chamber (16 h light: 8 h dark cycle at 28 °C). The 5-day-old seedlings were inoculated with rhizobia *Bradyrhizobium diazoefficiens* USDA110 suspension media (OD_600_ = 0.08) and, thereafter, watered with nitrogen-free Hoagland nutrient solution comprising the following components: 2.5 mM K_2_SO_4_, 2 mM MgSO_4_·7H_2_O, 1 mM KH_2_PO_4_, 0.15 mM FeCl_2_, 1.5 mM CaSO_4_·2H_2_O, 4.6 × 10^−2^ mM H_3_BO_3_, 9.1 × 10^−3^ mM MnCl_2_·4H_2_O, 7.5 × 10^−4^ mM ZnSO_4_, 5 × 10^−4^ mM CuSO_4_, 1.1 × 10^−4^ mM MoO_3_, 9.4 × 10^−5^ mM CoCl_2_·6H_2_O, and 500 μM (P-sufficient conditions: OP) or 5 μM (P-deficient conditions: LP) of KH_2_PO_4_ (pH = 5.8) once a week. Soybean plants and nodules were separately harvested at 28 dpi (days after inoculation) to measure fresh weight, dry weight, height of shoot, total P and N contents, nodule number, and nitrogenase and Apase activity.

For spatial expression analysis, nodules at different developmental stages were separately harvested at 10, 17, and 28 dpi. All tissues were frozen in liquid nitrogen and stored at −80 °C for further RNA extraction and qRT-PCR analysis.

Pot experiments in phytotron were conducted for the effect of overexpression of GmPAP4 on the seed yield of soybean plants. Selected soybean seeds were planted in ceramic pots (diameter: 45 cm; height: 30 cm; bottom diameter: 25 cm) containing vermiculite with a total of 10 pots prepared for each P treatment under optimal growth conditions. Fifty milliliters of rhizobia USDA110 solution (OD_600_ = 0.08) was applied to each pot with two uniform 5-day-old seedlings. In the meantime, the plants were watered with OP or LP nutrient solution as above once a week and the pod number, seed number and weight were determined at the soybean maturation stage (n = 20).

### 4.2. Plant mRNA Isolation and Quantitative Real-Time PCR (qRT-PCR)

Total mRNA was extracted using the RNAprep pure plant kit (Tiangen, Beijing, China) and the resulting cDNA was synthesized using a PrimeScriptTM RT reagent kit with a gDNA eraser (Takara, Otsu, Shiga Prefecture, Japan). Our qRT-PCR analysis was run on a CFX96™ real-time system (Bio-Rad, Berkeley, CA, USA) using the SYBR Premix EX TaqTM (Takara, Otsu, Shiga Prefecture, Japan). The specific primers used are listed in Table 1. The cycle threshold (CT) values of each sample were standardized to the reference housekeeping gene, *GmActin11*, and the relative fold change (FC) of gene expression was calculated based on the 2^−ΔΔCT^ method [31].

### 4.3. Histochemical GUS Staining Analysis

To elucidate the expression pattern of *GmPAP4* in soybean nodules, we cloned 2000 bp promoter sequences of *GmPAP4* upstream of the translation start codon ATG, fused with β-glucuronidase (GUS) reporter gene (p*GmPAP4*:GUS). The resulting construct was introduced into the soybean hairy roots by *Agrobacterium rhizogenes* K599 and the transgenic hairy roots were then inoculated with rhizobia *Bradyrhizobium diazoefficiens* USDA110 for nodule development. After 4 weeks of rhizobia inoculation, transgenic hairy roots and nodules were stained as described previously and captured with a light microscope (Olympus U-TV0.5XC-3, Tokyo, Japan) [14]. For GUS activity, total nodule proteins were extracted and incubated in a mixture containing 10 mM 4-methylumbelliferyl β-D-glucuronide (MUG; Sigma Chemical Co., St.Louis, MO, USA) for 1 h at 37 °C. The fluorescence product of 4-methylumbelliferone (4-MU) was monitored using a VersaFluor™fluorometer (Bio-Rad, Hercules, CA, USA) with excitation at 365 nm and emission at 455 nm.

### 4.4. Generation of Stable Transgenic Soybean Plants

A full-length CDS (coding sequence) of *GmPAP4* was cloned into a modified overexpression vector pCAMBIA3300 and the resulting construct was introduced into the *Agrobacterium tumefaciens* strain, EHA105, for stable transgenic soybean transformation using the variety Jidou12 as the background. The positive transgenic plants were identified by bar resistance and, then, the bar gene was amplified using bar-specific primers (Table 1).

### 4.5. RNA In Situ Hybridization Analysis

RNA in situ *hybridization* analysis was performed exactly as previously described [32]. The digoxigenin-labelled antisense and sense RNA probes for *GmPAP4* were 5′-DIG-AAAGAAGCGUGAGAAUCAGAACAAGAAGGAGU-3′ and 5′-DIG-ACUCCUUCUUGUUCUGAUUCUCACGCUUCUUU-3′, respectively. The hybridization signals were detected by alkaline phosphatase-catalysed color reaction with 5-bromo-4-chloro-3-indolyl phosphate/nitroblue tetrazolium (Roche; catalogue no. 11175041910, Basel, Switzerland).

### 4.6. Toluidine Blue Staining

Toluidine blue staining for the observation of infection cells in nodules was performed as described in our previous study [33]. The percentage of the area of infection cells to total cells in a nodule section and the surface area of 100 infection cells were calculated using Image-Pro Plus 6.0 software.

### 4.7. Measurement of N and P Contents

The measurements of total N and P contents were conducted according to our previous study [14]. Fresh plant samples were dried in an oven at 80 °C first and 0.3 g dried samples were ground and digested with concentrated H_2_SO_4_ in a microwave oven. The P content was measured by the color reaction of P-molybdate blue at the absorbance of 700 nm and the N content was determined using the semimicro-Kjeldahl determination method in a nitrogen analyzer.

### 4.8. Statistical Analysis

Means and SE values were calculated by using GraphPad Prism version 8.0.2 (263) (GraphPad Software Inc., San Diego, CA, USA). The two-tailed Student’s *t*-test was used to calculate the significance between the samples.

## 5. Conclusions

Overall, in our study, we characterized a purple acid phosphatase, *GmPAP4*, that was specifically expressed in infected cells of soybean nodules and it could be induced by low P stress. Functional analysis showed that overexpression of *GmPAP4* greatly increased nodule number and nitrogen fixation activity, thus enhancing the acquisition of nitrogen and phosphate under low P condition and, as a consequence, enhancing the seed yield of soybean. This study provided a new gene with a high nitrogen-fixing ability for soybean molecular breeding.

## Figures and Tables

**Table 1:** All primers used in this study.

Primer Name	Sequence (5′ to 3′)
GmPAP4-RT-F1	GAGCCGTTTGCGAGTACAAG
GmPAP4-RT-R1	TCTGCATAGGAGCCAAGCAT
GmActin11-F	ATCTTGACTGAGCGTGGTTATTCC
GmActin11-R	GCTGGTCCTGGCTGTCTCC
Bar-F	CCATCGTCAACCACTACATCGAGACA
Bar-R	CTTCAGCAGGTGGGTGTAGAGCGT

## Data Availability

Data is contained within the article and Supplementary Materials.

## Supplementary Materials

The following supporting information can be downloaded at: https://www.mdpi.com/article/10.3390/ijms25073649/s1.
