# Supplementary material for: Overexpression of GmPAP4 Enhances Symbiotic Nitrogen Fixation and Seed Yield in Soybean under Phosphorus-Deficient Condition
Source: Int J Mol Sci. 2024 Mar 25;25(7):3649. doi: 10.3390/ijms25073649 (PMC11011270; doi:10.3390/ijms25073649)
Supplement: Supplementary file 1 [file ijms-25-03649-s001.zip › ijms-2889915-supplementary.pdf]

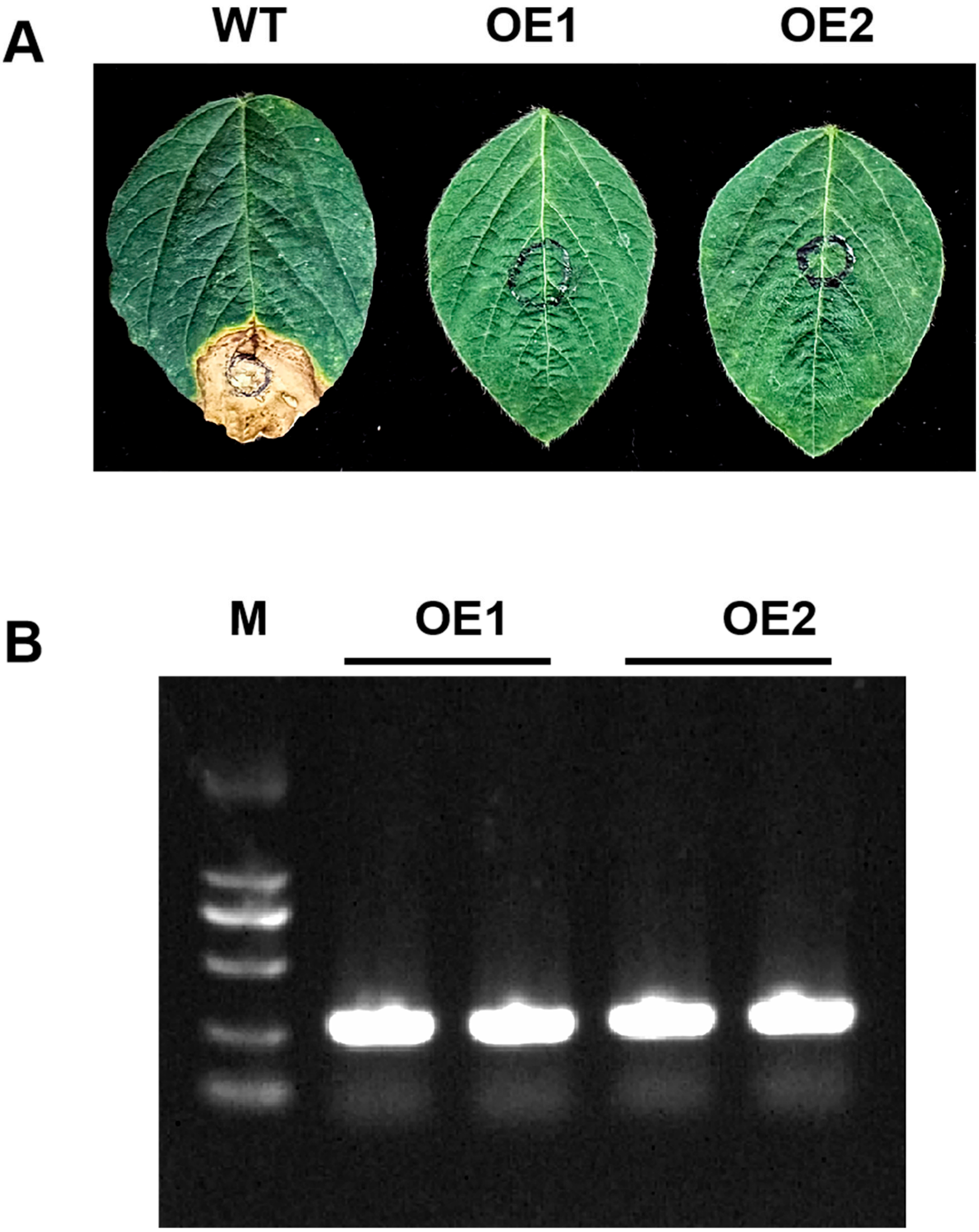

Figure S1. Identification of positive stable transgenic *GmPAP4* OE lines. A. Identification of positive transgenic OE lines through glyphosate resistance detection. B. Identification of positive transgenic OE lines through PCR targeting bialaphos resistance gene.

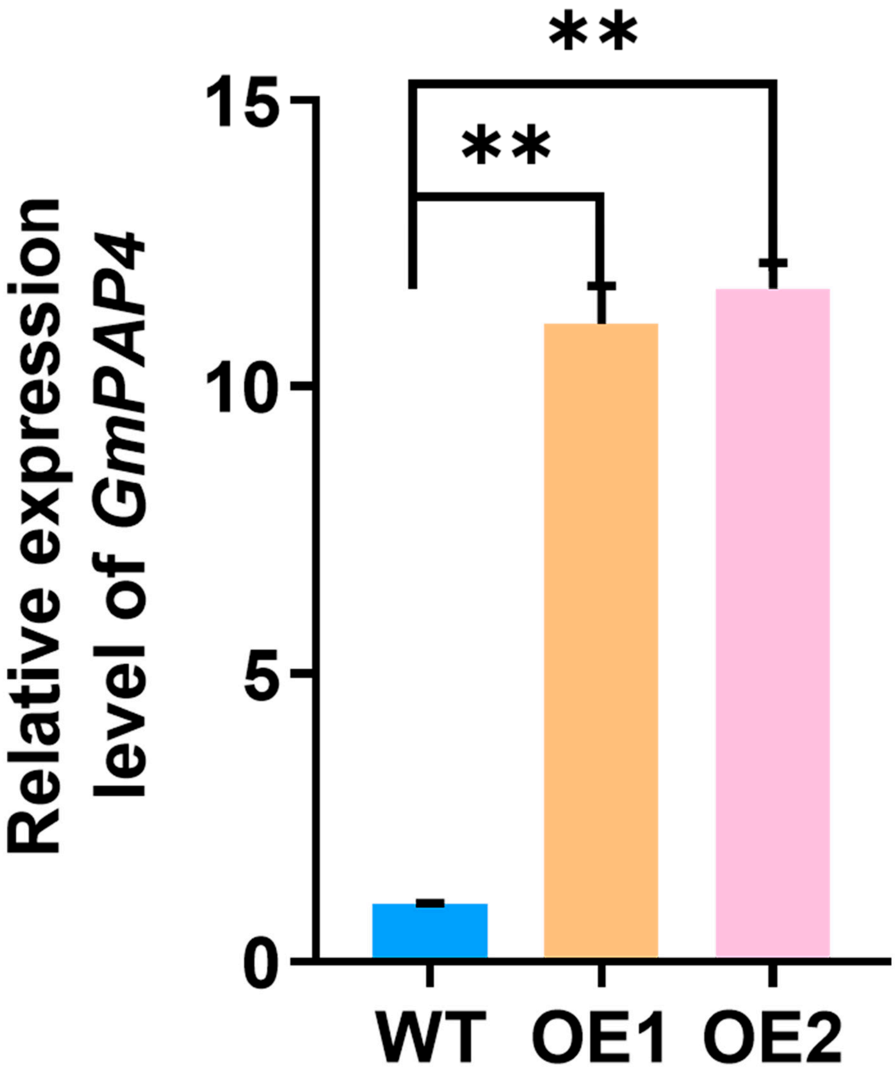

Figure S2. Relative transcript accumulation of GmPAP4 in nodules of OE lines at 28 dpi.

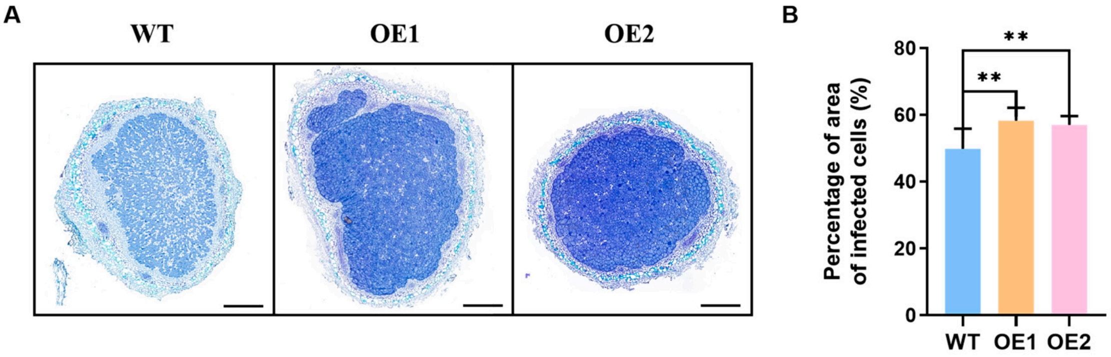

Figure S3. Interior structural characteristics of nodules formed on *GmPAP4* OE lines. (A) Toluidine blue staining of nodules. (B) Percentage of area of rhizobia-infected cells to all cells in one nodule section. (A) Scale bar =500μm. Asterisks indicate statistically significant differences according to Student's t-test (two-tailed) (**P< 0.01).
